# Supplementary figures and images for: Integrative Transcriptomics and Proteomics Analysis Reveals Immune Response Process in Bovine Viral Diarrhea Virus-1-Infected Peripheral Blood Mononuclear Cells
Source: Vet Sci. 2023 Sep 28;10(10):596. doi: 10.3390/vetsci10100596 (PMC10611041; doi:10.3390/vetsci10100596)

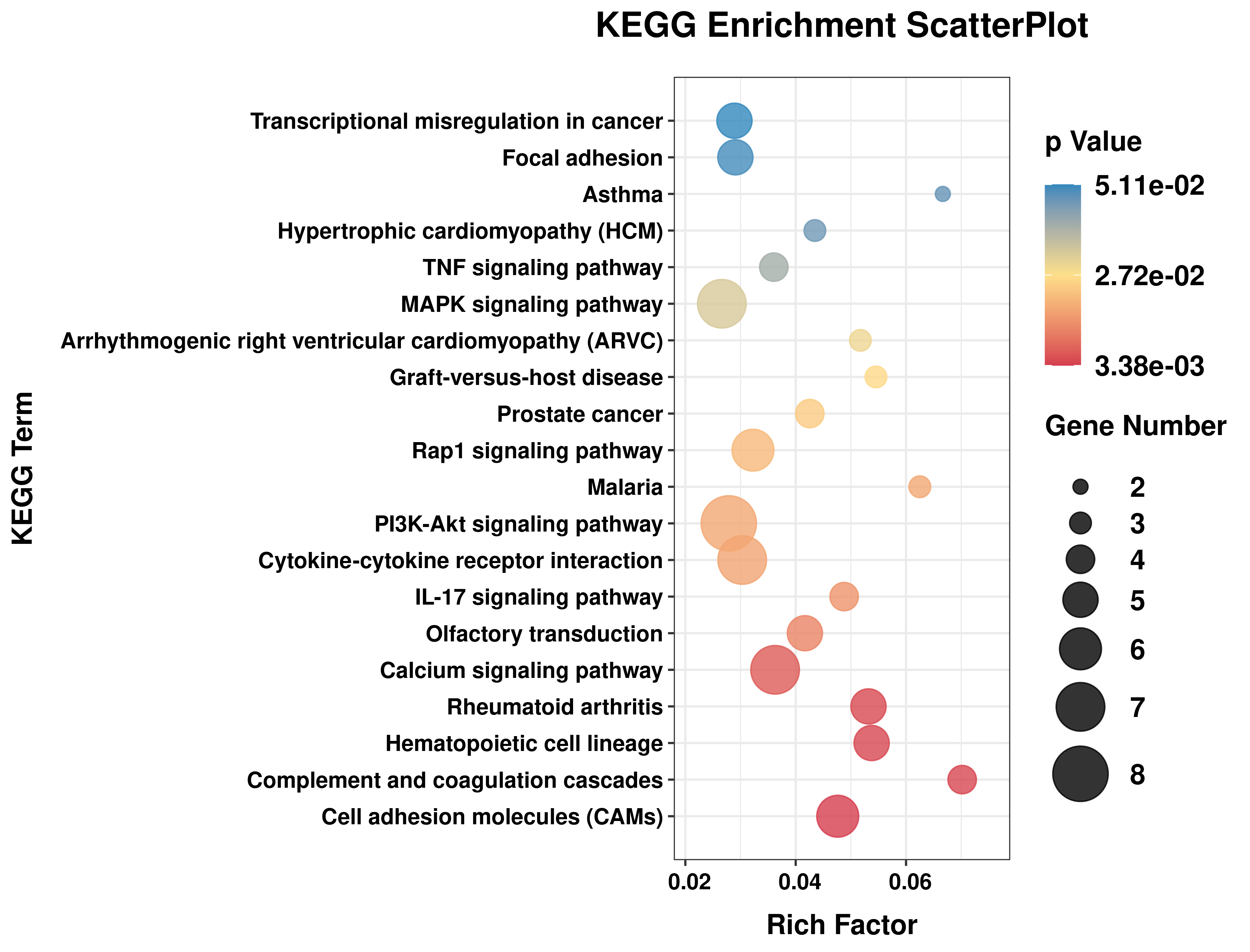

Supplement: Supplementary file 1 [file vetsci-10-00596-s001.zip › vetsci-2606959-supplementary/Supplemntary figure 1.png]

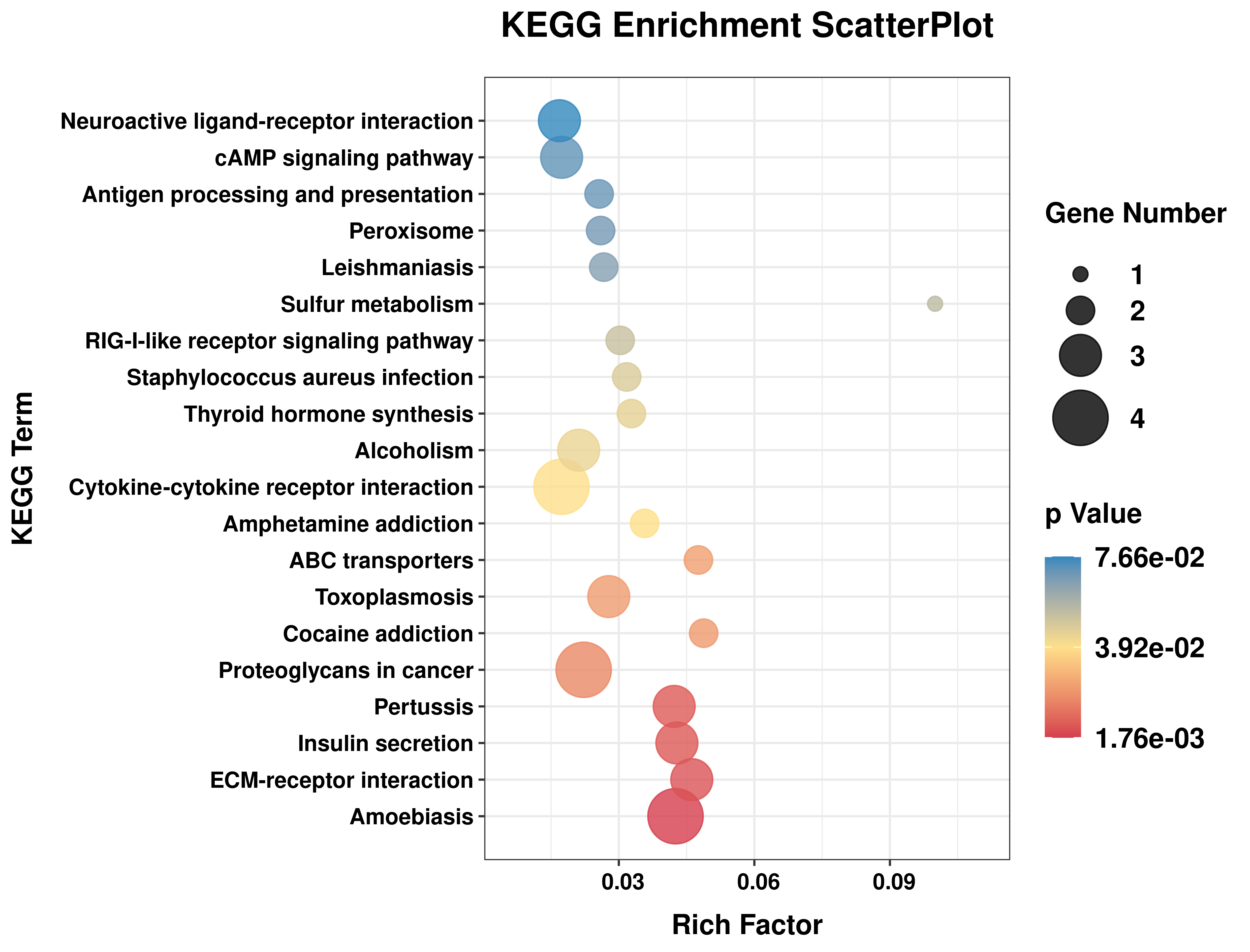

Supplement: Supplementary file 1 [file vetsci-10-00596-s001.zip › vetsci-2606959-supplementary/Supplemntary figure 2.png]

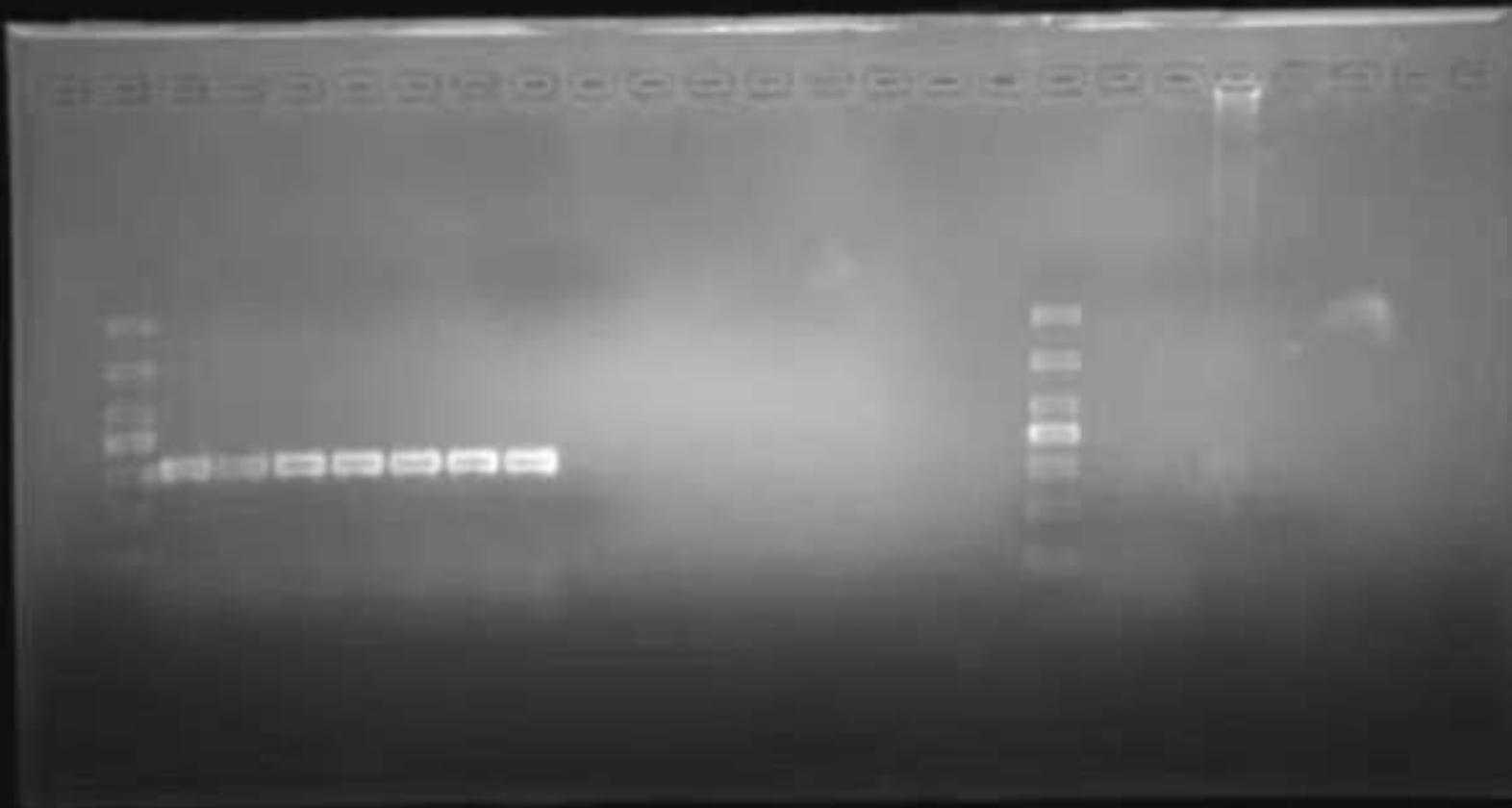

This original image is for figure1A in manuscript

Supplement: Supplementary file 1 [file vetsci-10-00596-s001.zip › vetsci-2606959-supplementary/vetsci-2606959-original figure.pdf]
